# The Effect of Nanobubbles on Transdermal Applications

**DOI:** 10.3390/nano13182600

**Published:** 2023-09-20

**Authors:** Athanasios Ch. Mitropoulos, Christina Pappa, Ramonna I. Kosheleva, George Z. Kyzas

**Affiliations:** Department of Chemistry, International Hellenic University, Kavala Campus, 65404 Kavala, Greece

**Keywords:** nanobubbles, skin penetration, biomimic membrane, transdermal absorption

## Abstract

In the present work, a new method for dermal delivery using nanobubbles (NBs) is investigated. Oxygen NBs are generated in deionized water and used to produce cosmetic formulations with hyaluronic acid as an active ingredient. Nanobubbles result in the improvement of the effect and penetration of the active ingredient through Strat-M, a synthetic membrane that resembles human skin. Experiments conducted with the Franz Cell device confirm the greater penetration of the active ingredient into Strat-M due to NBs, compared to cosmetic formulations that do not contain NBs. The effect of NBs was further examined by measuring UV-Vis and FTIR spectra. A possible mechanism was outlined, too. It was also found that NBs do not change the pH or the FTIR spectrum of the cosmetic serum indicating non-toxicity.

## 1. Introduction

People have used different formulas on their skin for thousands of years to achieve a range of therapeutic or cosmetic results [1,2,3]. Penetration of the valuable active ingredients through the skin is preferred compared to other invasive methods which are laborious, time consuming, costly and may cause various side effects. However, the primary physiological barrier that defends humans and other mammals’ bodies from outside diseases and physical and chemical attacks is their skin [4,5,6,7]. This skin’s barrier function is a crucial one to the penetration of active ingredients and limits their delivery amount [8,9,10,11]. To address these restrictions by increasing the penetration effect, formulations are continually being developed, such as hydrogels, nanoemulsions, micelles, and liposomes. [12,13,14]. However, the substances frequently added to formulations to increase penetration are biologically hazardous and may irritate the skin [15,16]. The use of additives, such as chemical boosters, is the most popular and practical way to get around this barrier function. These substances could “piggyback” the actives into the Stratum Corneum (SC) or alter the structure of the SC to make it more permeable to the actives [1]. Most studies on chemical penetration enhancers to far have focused on their use in formulations to alter the permeability of different active substances. Using nanocarriers as drug or ingredient carriers may improve the therapeutic efficacy. Additionally, delivery using nanoparticles at the inside the skin can improve drug and ingredient retention with controllable release kinetics [17]. It has been documented that nanoparticles can enter the skin by the trans appendageal route, which passes through the sebaceous and pilosebaceous glands, sweat glands, and hair follicles [18,19]. This makes it possible for nanoparticles to enter the stratum corneum, the skin’s outermost layer of defense. New dermal delivery techniques with minimal or no chemical use are required to get beyond these barriers.

The various physical and chemical properties of tailored nanoparticles used in cosmetics have led to the development of several innovative and useful applications to improve our lives. However, these same characteristics have also made it possible for them to be incorporated into the human body and work in unique ways with biological systems. As a result, nanotoxicity is now a legitimate concern, and research in nanotoxicology has grown recently [20].

Despite our common awareness that such penetration levels are little, permeation in the skin with the aid of topical skin treatments is one of the many potential ways of nanoparticles entry into the body that warrants attention. The impact of skin types and the physical skin barrier’s integrity on the permeation of nanoparticles has not been adequately studied.

Due to their unique features, Nanobubbles (NBs), which are described as submicron-scale gas cavities, have drawn more attention in the field of medication delivery [21,22,23]. First, NBs are fully clear solutions and are invisible to the naked eye due to their diameter being smaller than the wavelength of visible light [24,25]. Second, due to higher Brownian motion than buoyancy, NBs are extremely stable in the solution for several months [26,27]. Third, NBs can store the active substances at their interface with the solvent due to their extremely large surface area [28,29]. NBs with these distinct qualities have been applied in a variety of sectors as mediators for the delivery of active substances, such as medicine and healthcare, agriculture, food, environmental remediation, water treatment, having great advantages [30,31]. Although NBs seem to have many potentials in the field of skin penetration in pharmaceutical and cosmetic industry, the effect they may have on the skin and possible irritation during their administration as well as the mechanism of penetration have not yet been studied.

Younger skin has a lot of hyaluronic acid (HA), an endogenous glycosaminoglycan, but as skin ages, this amount naturally diminishes. HA is commonly used as a moisturizing agent because of its capacity to hold onto water molecules. In addition, as HA is a natural component of the extracellular matrix of the skin, it has anti-aging properties. Despite these advantageous effects, the huge MW (>500 kDa) and high hydrophilicity of HA cause poor penetration, which severely restricts its potential [32,33,34]. HA can be utilized as a soft-tissue filler as well as incorporated into a variety of topical cosmetic treatments. In this instance, intradermal microinjection is used to overcome the low permeability of HA [32]. Although maximal accumulation down to the dermal layer is possible with HA microinjection, which accounts for 80% of filler treatments in the US, numerous non-invasive ways have been proposed to enhance HA performance, some of which take advantage of the usage of nanotechnology. For instance, Jegasothy et al. believed that by lowering the MW of HA and forming the polymer as NPs (nano-HA, 5 nm), a higher penetration of HA may be accomplished [35,36]. Although the efficacy of products based on nano-HA was shown, this study did not compare the outcomes to those of non-nanosized HA treatments.

HA nanoformulation has been investigated as an alternate method for boosting penetration in addition to size reduction. In order to assess the ex-vivo penetration of free HA and HA NPs in full-thickness mouse skin, Tokudome et al. produced a 100 nm complex consisting of HA (1200 kDa) and cationic protamine [35,36]. Chen et al. employed skin-penetrating peptide (SPACE) conjugation to enhance the functionality of HA-loaded ethosomes, which was a distinct strategy [35,36,37]. A greater HA penetration was seen with the conjugated ethosomes as compared to free-HA and non-conjugated NPs when the efficacy of the formulation was assessed in vivo (in hairless mice) and ex vivo (using porcine and human skin). To get around restrictions on moving hydrophilic macromolecules across skin layers, Martins et al. created a nanodispersion employing lipid materials. The complex between the surfactant and protein coupled with the HA was lyophilized and dispersed in isopropyl myristate by ultrasonication, creating a S/O nanodispersion. The authors employed a solid-in-oil (S/O) method [37,38]. On abdominal pig skin, an NP formulation containing 30 kDa HA was evaluated in vitro and demonstrated effective penetration across the SC layer. The findings revealed that the dissociation of the nanocomplex was the cause of the diffusion of HA into the dermis [38].

In this work, NBs are used in the production of cosmetic formulations as a new technique for dermal delivery of active ingredients. For this purpose, NBs contained in deionized water (DIW) are utilized, and the efficient administration of the active components is investigated. The NBs are created in a NB-generator [1,39] by supplying oxygen gas in a simple process and then the water containing the NBs is used in a simple cosmetic product without the need for any additional chemicals or complicated chemical processes. Utilizing the Franz Cell method, the release effect of the active components was investigated after cosmetic preparation on the Strat-M membrane of Merck which is a reliable alternative for the study of skin penetration [40,41,42]. The size of the NBs is measured over time by Nanoparticle Tracking Analysis and their effect on Strat-M is examined by UV-Vis and FT-IR spectrophotometers. Cosmetics containing NBs are compared with corresponding cosmetics which do not contain NBs. If our hypothesis is valid, an increase in the penetration of the active ingredients is expected.

## 2. Materials and Methods

### 2.1. Method of Preparation NBs

Water electrolysis, cavitation and other mechanical ways can lead to the formation of NBs [43,44,45]. In the present study NBs were created by hydrodynamic cavitation. According to Bernoulli equation (Equation (1)):

(1) $$P+\frac{\rho U^{2}}{2}=C$$

where *P* (Pa) is the pressure, *U* (m^3^/s) is the water flow rate at some point, *ρ* (kg/m^3^) is the density of the liquid, and *C* is a constant.

The pressure becomes negative when the water flow rate is greater than the square root of 2*C*/*ρ* and this marks the initiation of cavitation [46]. When a gas/liquid mixture flows through a Venturi tube during hydrodynamic cavitation with a decrease and subsequent increase in local pressure, bubbles are created. In this way a high number density of NBs can be regulated by controlling the liquid and gas flow rate through the venturi injection with the use of a mass flow meter.

**Figure 1:**
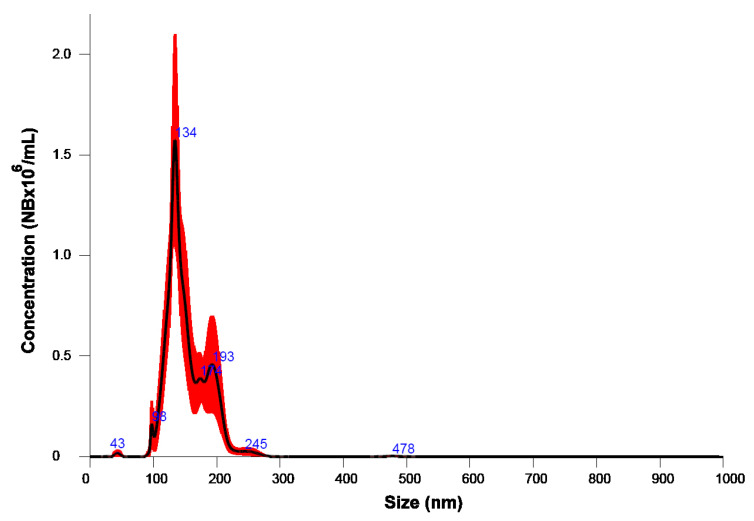
Size of NBs in water (the red band shows errors).

For the needs of this work NBs in DIW were produced by channeling O_2_ with a gas supply *Q* = 1.2 L/min for 15 min. Figure 1 shows the size of the generated NBs against their concentration with the specific operation parameters. The mean size was 151.5 ± 6 nm and the concentration 67.2 × 10^6^ ± 5 × 10^6^ NB/mL. In general, size as well as concentration of NBs scatters from time to time. Since our study lasted more than two months, it was concluded that the size polydispersity of NBs does not critically influence their penetration through the SC. This could be possible if their size had changed from nano- to micro-bubbles; which is not the case in this work.

### 2.2. Experimental Materials

Various materials were used to create two different cosmetic products. More specifically, a cosmetic serum was created that contains hyaluronic acid aqueous solution 1% as an active ingredient, in one case with DIW and in the other with DIW containing O_2_-NBs; the differences between them were compared. The raw materials were used for the cosmetic preparations are given next.

Hyaluronic acid solution 1% in water (HA): (INCI: Sodium Hyaluronate-Aqua) by Biothech Co., Ltd., Shandong, China, under the trade name Clear HA-100 with molecular weight 1400–1800 kDa. The use effect is related to the molecular weight of the product. High molecular weight: forms a dense protective film, provides long-lasting hydration, and has a good regenerative effect. Medium molecular weight: good moisturizing and lubricating effect, slow release and stable emulsifying effect. Low Molecular Weight: provides skin-nourishing benefits and long-lasting hydration. The percentage of high molecular weight hyaluronic acid is 35–45%, the medium molecular weight is 15–25% and the low molecular weight is 35–45%.

Vegetable glycerin: (INCI: Glycerol—Glycerin by Ambrogio Pagani S.p.a., Bergamo, Italy, with trade name Glycerin 99.5% Pharma 1,2,3-Propane-triol. Glycerol is found in all natural lipids, animal, or vegetable.

Xanthan gum: (INCI: Xanthan gum) by Kahl GmbH & Co. KG, Trittau, Germany, under trade name 6650 Kahlgum FQ80. High molecular weight polysaccharide known as xanthan gum is created through fermentation. Its main functional property is its ability to control the rheology of aqueous systems.

### 2.3. Formulation

The creation of the serum with HA solution 1% was carried out in two series of experiments to determine the impact of O_2_-NBs. The first series of experiments was performed by using DIW 92%, vegetable glycerin 3.5%, xanthan gum 0.5% and HA 4% (in aqua solution 1%), while the second series of experiments was performed by using DIW with O_2_-NBs 92% and the remaining materials in the same quantities. Cosmetic formulations of 100 g were created by mechanical agitation in an RSLab-13 digital shaker with a 5 × 40 cm SS cross fitting and operated at 1350 rpm.

### 2.4. Analytical Methods

Strat-M Membrane was used to the Franz Cell (Size 25 mm discs, Millipore Ltd., Tullagreen, Carrigtwohill, Co Cork IRL, Ireland). Strat-M membranes are commonly used for in vitro permeation studies and are designed to mimic the barrier properties of human skin. The Franz cell, which was produced from Permegear.de (Franz Cell in clear jacket, 15 mm, with a flat ground joint, 12 mL receptor volume), was used to measure the permeation or diffusion of active ingredients. Between the donor and receptor chambers of the Franz cell, a Strat-M membrane was installed. NaCl aqueous solution (0.9% by Bioser S.A., Trikala, Greece) was poured into the receptor chamber. The donor chamber was supplied with the cosmetic serum (1 mL). One mL aliquots were taken out of the receptor chamber at 30 min, 1 h, 2 h and 3 h, and the equivalent volume of NaCl aqueous solution (0.9%) was added after extraction. More analysis was done on the collected aliquots. The experimental conditions are ambient temperature and atmospheric pressure each time.

The ultraviolet-visible (UV-Vis) spectrophotometer used in the measurements was the Shimadzu UV-1900i. (Europa GmbH, Duisburg, Germany). Quartz cuvettes were used and the absorbances of the samples at the maximum wavelength were measured. Deionized water and DIW with O_2_-NBs were used for sample dilutions. The samples taken from Franz Cell were diluted 1:2 with the NaCl aqueous solution (0.9%) to allow them to be placed in the cuvette (small sample volume). Sodium chloride aqueous solution (0.9%) was used as a blank and the spectrometer was zeroed.

The size distribution and concentration of the particles in liquid suspension were determined using Nanoparticle Tracking Analysis (NTA; NanoSight LM10, Malvern Instruments, Malvern, UK). NTA: The same samples taken from Franz Cell analyzed at NTA. NaCl aqueous solution (0.9%), DIW, DIW with O_2_-NBs and HA were also analyzed.

Using a Zetasizer Nano ZS ZEN3600 (Malvern Instruments Ltd., Worcestershire, UK), cosmetic samples were evaluated for particle surface potential (zeta potential). The Zetasizer Software version 6.30 was used to calculate the surface charge (zeta potential) characteristics for all the measurements, which were all carried out at 25 °C. Phase analysis light scattering (PALS) and laser Doppler velocimetry are the two techniques that make up the measurement.

PerkinElmer’s FT-IR spectrometer is used to confirm production materials (incoming or departing) and identify unidentified materials. Most of the time, the information is highly specific, allowing for subtle differences between materials that are comparable. Strat-M membranes after Franz Cell experiments were analyzed at the FT-IR Spectrometer from the smooth (polyether sulfone side) and rough (polyolefin) side. As reference an unused Strat-M membrane was analyzed. The liquid cosmetic serum samples were also analyzed.

Auxiliary equipments are used too. Kern ABT analytical balance (model 220-5DM, weighing capacity 82–220 g, AC/DC input 220–240 V AC, universal plug set) used for weighing both the materials and the membranes. Multiparameter pH Meter Hanna Edge HI2020-02 used for measuring pH, conductivity, and dissolved oxygen through its unique digital electrodes.

## 3. Results and Discussion

**Figure 2:**
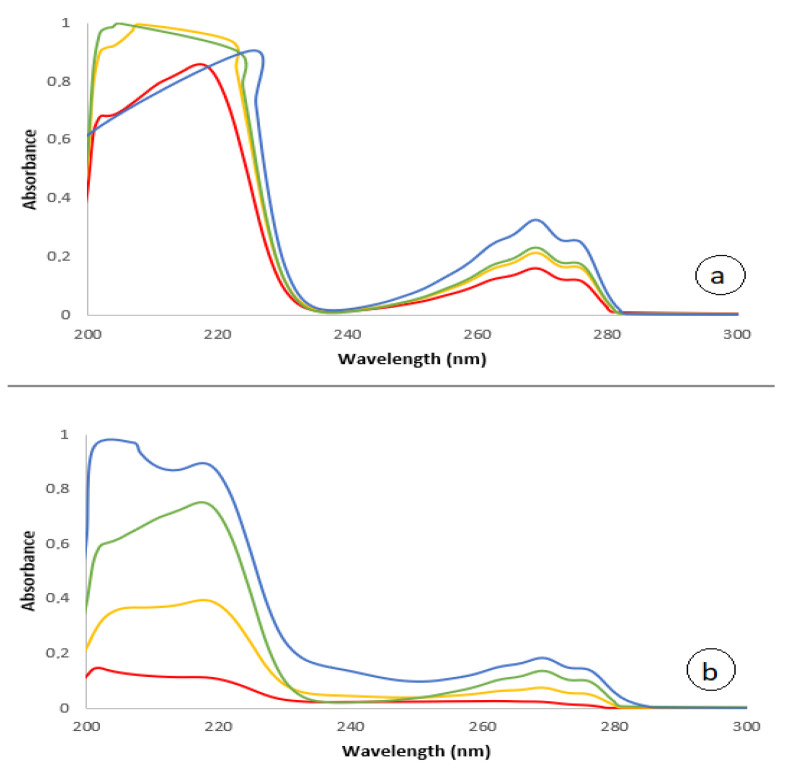
UV-Vis spectra: (**a**) HA in DIW without NBs; (**b**) HA in DIW with O_2_-NBs. Red line after 30 min; yellow line after 1 h; green line after 2 h; blue line after 3 h.

From the Franz Cell experiments, aliquots (1 mL) were extracted from the receptor chamber at 30 min, 1 h, 2 h and 3 h. Measurements of samples taken from the Franz Cell are analyzed in the UV-Vis spectrophotometer at a 1:2 dilution for the serum containing HA [47]. Figure 2 shows the result. A broad peak at 200–220 nm and a peak at 270 nm can be seen indicating the presence of HA in the samples [48]. The result indicates that an amount of HA, included in the cosmetic formulation with NBs, is absorbed in 30 min by the Strat-M membrane, whereas the same amount of HA included in the cosmetic formulation that does not contain NBs is absorbed in 3 h, whereas in 30 min only a minimal absorption is seen. A similar behavior is also observed by using niacinamide as an active ingredient where product was not changed using NBs [49].

**Figure 3:**
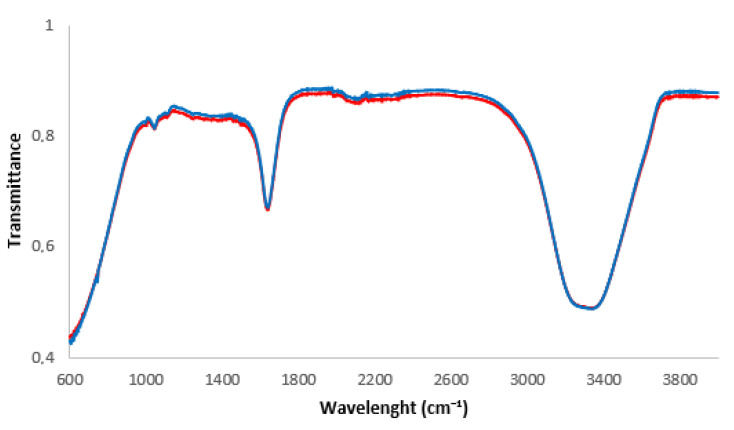
FTIR spectrum of serum with HA in DIW: Blue line without NBs; red line with NBs.

The samples were further analyzed by FT-IR [50] and the spectra appear to be identical (Figure 3). The cosmetic formulation due to NBs seems not to cause any toxicity or irritating and oxidizing. This conclusion derives from the fact that (i) FT-IR spectra of serum with HA and O_2_-NBs does not present any shift referencing to that without NBs and (ii) the measured pH of the liquid cosmetic serum, for both cases, is equal to 5.6. Taking all the above into the account, it was inferred that NBs accelerate the passage of the active ingredient through the Strat-M without any chemical complications.

**Figure 4:**
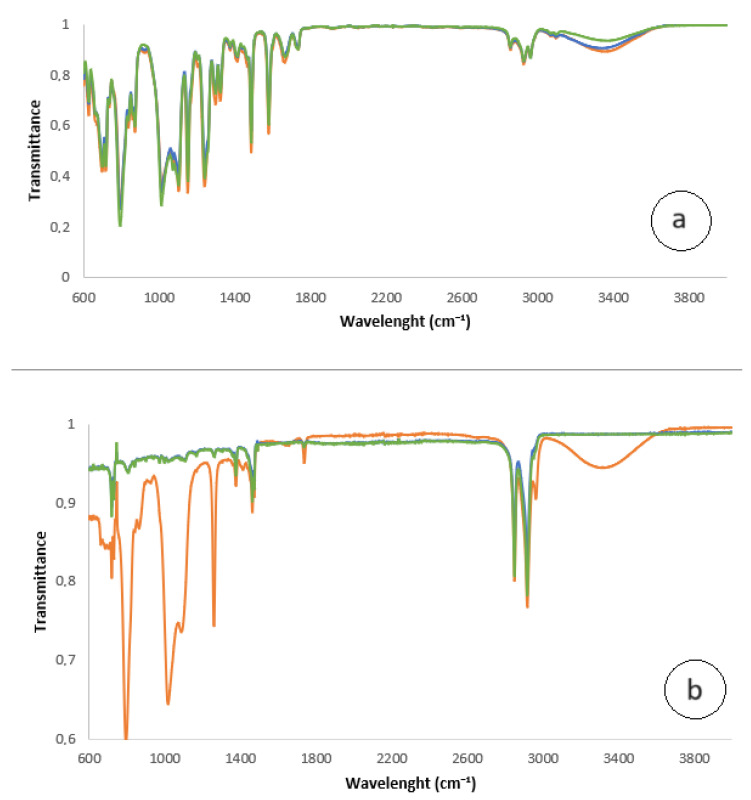
FTIR spectra of Strat-M. (**a**) smooth side; (**b**) rough side. Green line intact Strat-M membrane; blue line Strat-M with cosmetic serum and HA in DIW; orange line Strat-M with cosmetics serum and HA in DIW with NBs (see text for details).

The Strat-M membranes after Franz Cell experiments were analyzed at the FT-IR Spectrometer from the smooth and rough side. As reference an unused Strat-M membrane was analyzed, too. Figure 4 shows the spectra. On the smooth side the peaks appear to be similar, with the exception of a slight variation in the peak at 3370 cm^−1^, indicating the presence of hydroxyls and hydration [51]. On the rough side however, there are differences between the two cases. There are three more peaks at the spectrum of the membrane with NBs cosmetic serum at 794, 1019–1091 and 1260 cm^−1^ and a distinct appearance of a broad peak at 3370 cm^−1^.

At 1260 cm^−1^ the peak is often associated with vibrations involving C-N bonds. This could include stretching vibrations of C-N bonds in primary amines, as well as vibrations in nitriles (C≡N) or amides (C=O-NH) functional groups [52]. The peak at 3370 cm^−1^ is a telltale that hydration when NB are present have reached at the lower level of the Strat-M membrane that resembles the subcutaneous fatty tissue [51].

**Figure 5:**
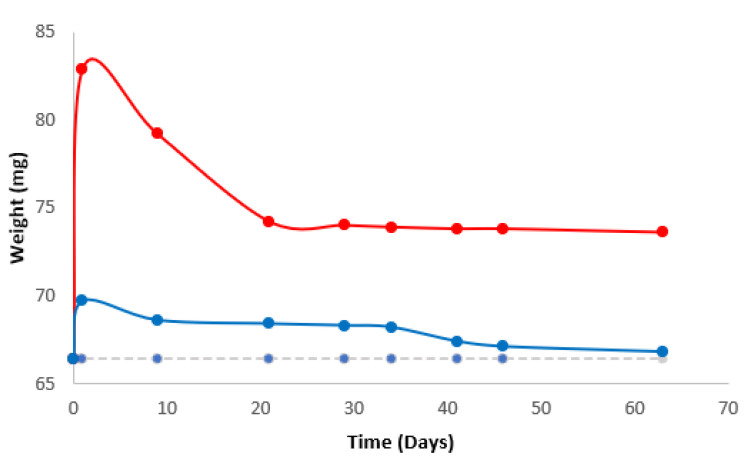
Weight of Strat-M. Broken line intact Strat-M; blue line Strat-M with cosmetic serum and HA in DIW; red line Start-M with cosmetic serum and HA in DIW with NBs.

By weighting the membranes before and after the Franz Cell experiments it was found that the membrane with NBs after a period of 63 days remains hydrated and the cosmetic product has not completely disappeared from it, as seems to be the case with the membrane with DIW without NBs, which tends to reach the weight of the reference membrane from the 10th day. Figure 5 shows the result. In the case of NBs the weight of Strat-M increases by 25% in the first day and after 20 days decays to 11% and remains there for 2 months. In the absence of NBs the weight increases by 5% and then constantly decays to less than 1% after 2 months. Since the product penetrates the lower layer of the epidermis, it is not affected by any skin washing during this time.

**Figure 6:**
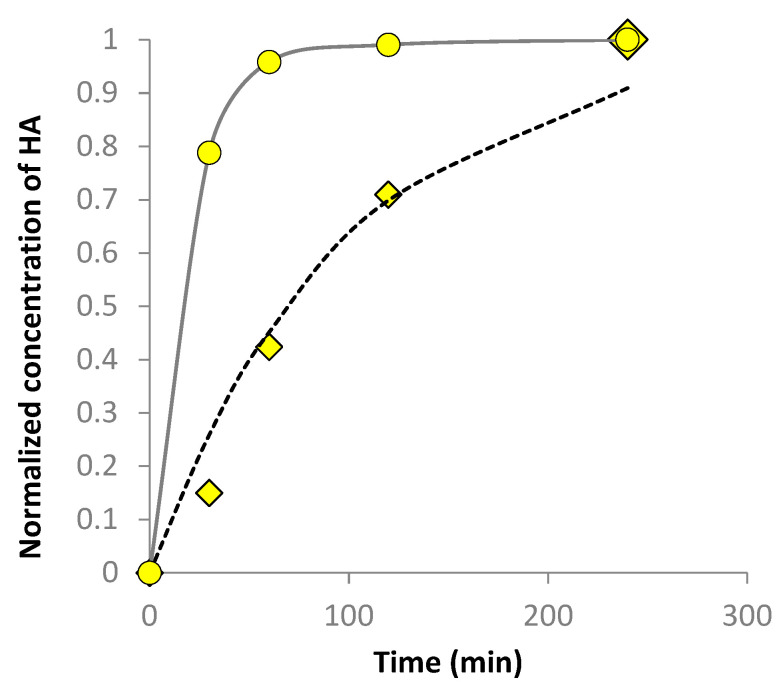
Franz cell serum permeation curves. Circles; HA in DIW with O2-NBs. Diamond; HA in DIW without NBs. Dushed black line corresponds to first order kinetic equation.

Figure 6 shows the results from serum permeation though Strat-M during Franz cell experiments. The data are withdrawn from UV-Vis spectra at different sampling times. In the case of serum without NBs, the experimental values follow a first order kinetic model, as it was expected according to the literature [53,54].

(2) $$\frac{Q}{Q_{O}}=1-e^{-kt}$$

where, *Q*/*Q_o_* is the concentration of HA (normalized), *t* is the time in minutes and *k* the kinetic constant *k* = 0.01 (1/min). On the other hand, the kinetics of HA diffusion through the membrane with the presence of NBs demonstrates a gamma shape curve suggesting very fast kinetics, reaching the maximum of HA in 60 min.

**Figure 7:**
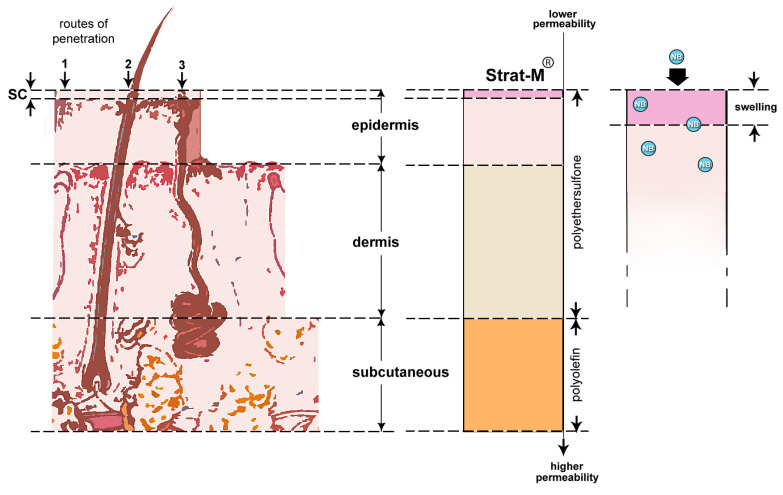
Regions of human skin versus Strat-M membrane. Left (human skin regions): top layer epidermis with penetration routes (1) Stratum Corneum (SC), (2) hair follicles, and (3) sweat glands; middle layer dermis; bottom layer subcutaneous fatty tissue. Middle (Strat-M): top layer made by polyethersulfone having lower permeability; bottom layer made by polyolefin having higher permeability. Right (Start-M): Swelling of the top layer after Interaction with NBs.

Figure 7 shows the 3 layers of human skin: epidermis, dermis, and subcutaneous fatty tissue. The stratum corneum (SC) is the epidermis’ outer layer. There are 3 routes for an active ingredient to penetrate SC: (1) via the horny layer, which is the dominant route, (2) through hair follicles, and (3) through sweat glands. Via SC there are two pathways for delivering active ingredients: the intercellular and the intracellular. The former is of prime interest in this work. According to Barry [53], increasing the volume of the polar zone and hence the cross-sectional area accessible for polar diffusion is one strategy to improve the penetration of the active component through the stratum corneum to the live tissue. Increased hydration does not, however, result in the expansion of this region, according to X-ray studies.

On the other hand, since NBs have a negative charge, they can assist expansion of the polar region and the resulted swelling can provide a larger fractional volume. Strat-M membrane has multiple layers each one having different permeability [23]. The upper layer is made by polyethersulfone and resist to diffusion whereas the lower layer is made by polyolefin and is more permeable. Figure 6 depicts the possible mechanism of NBs on Strat-M layers. This is a similar mechanism when enhancers and accelerators such as DMSO are applied to boost the active components’ uptake through SC’s intercellular route [55].

Zeta potential is a key parameter used to assess the stability of cosmetic formulations, such as creams, lotions, and suspensions. It indicates the electrostatic repulsion or attraction between particles in the formulation. Higher absolute zeta potential values generally indicate better stability, as strong repulsive forces prevent particles from aggregating and settling [56]. In addition, zeta potential is related to other quality properties of cosmetic products such as texture and feel and ingredient delivery according to application [57,58]. In general, any colloid system with zeta-potential above ±30 mV is considered to be very stable. Specifically, for cosmetic products it is desirable to have a zeta-potential close to the human skin (about −40 mV [59]). In the present case, the zeta potential measurements indicate that NBs does not impact the stability of the formulation showing comparable values for serum with NBs −57.1 mV and without NBs −66.8 mV. Another factor that interplays with zeta-potential is pH; a change in pH alters zeta-potential and vice versa. This is in accordance with our result that NBs does not alter the pH values of the examined formulation [60].

## 4. Conclusions

Cosmetic formulations containing O_2_-NBs have better and faster penetration into the skin than the corresponding cosmetics without NBs. Regarding the serum with hyaluronic acid, the same amount of active substance passed through the membrane for the product with O_2_-NBs in 30 min, requires 3 h for the product without NBs. In fact, within the first 60 min, the total of HA passed the membrane and the mechanism seems to be different, forming a gamma shape curve while the case without NBs follows a first-order equation. Moreover, the use of NBs permit a long lasting of active ingrdients and skin hydration of more than 2 months whereas water alone looses them in a few days as concluded by weight measurements of the membranes used. From experiments in the Franz Cell combined with FT-IR spectra, it was found that the NBs remain in the lower layers of the membrane and are retained.

A possible mechanism for the interaction of NBs with the membrane was outlined. Nanobubbles act as enhancers allowing a sweling of the polar resion of Strat-M and therefore increasing polar diffususion. The use of water alone (i.e., without NBs) does not widen this region as already been known from X-ray studies on human SC conducted by others. Therefore, NBs act as chemical enhnacers without causing skin irritation and oxidization as pH measurments and FTIR spectra suggest.

Nanobubbles is a rather new field in nanotechnology and the exact mechanism for their surviving has not yet determined. The use of NBs as substitute of chemical enhancers for skin penetration requires further studies with different products, cosmetic and pharmacuticals. To this end there will be some future work.

## Data Availability

The data presented in this study are available on request from the corresponding author.
